# 液相色谱-串联质谱法与气相色谱-质谱法测定毛发中15种芬太尼类似物和苯丙胺类兴奋剂

**DOI:** 10.3724/SP.J.1123.2025.12005

**Published:** 2026-09-08

**Authors:** Qi WEI, Fuhai SU, Xin ZHU

**Affiliations:** 中国计量科学研究院，北京 100029; National Institute of Metrology，Beijing 100029，China

**Keywords:** 芬太尼类似物, 苯丙胺类兴奋剂, 毛发, 标准物质, 液相色谱-串联质谱, 气相色谱-质谱, fentanyl analogs, amphetamine-type stimulants, hair, reference material, liquid chromatography-tandem mass spectrometry （LC-MS/MS）, gas chromatography-mass spectrometry （GC-MS）

## Abstract

药物滥用，尤其是芬太尼类似物及苯丙胺类兴奋剂（ATS）等合成毒品的泛滥，已成为全球重大公共卫生问题。毛发分析能回溯数年的用药史，在司法鉴定领域独具优势，但其应用受限于基质有证标准物质的缺乏。本研究基于水-二甲基亚砜-盐酸混合溶液浸泡法，以15种目标药物（9种芬太尼类似物和6种苯丙胺类兴奋剂）为研究对象，建立了毛发样本的制备及液相色谱-串联质谱法（LC-MS/MS）与气相色谱-质谱法（GC-MS）双平台定量分析方法。实际制备结果表明，除苯丙胺（AP）外的14种药物均成功掺入毛发基质。将该双平台技术应用于该类基质标准物质的定值分析，通过双平台交叉验证提升了定值结果的可靠性，为该类标准物质的研制提供了准确可靠的定值手段。采用Box-Behnken响应面法优化前处理条件，优化后的提取条件如下：提取温度40 ℃，提取时间50 min，液固比100∶1（mL/g），盐酸浓度0.01 mol/L。结果表明，制备的毛发样本中14种目标物的含量为1.050～7.771 ng/mg，日内与日间精密度（相对标准偏差<10%）、回收率（84.46%～116.4%）及基质效应（80%～120%）均满足分析要求。两种方法的灵敏度均较高，LC-MS/MS的检出限和定量限分别为0.05 pg/mg与0.25 pg/mg，GC-MS的检出限与定量限分别为0.02 ng/mg和0.08 ng/mg。通过*F*检验验证，在显著性水平0.05（自由度为10，22）下，毛发样本均匀性良好。本研究建立了基于LC-MS/MS与GC-MS的双平台平行检测与交叉验证策略，通过对同一样本独立测定与结果比对，实现了结果的内部交叉验证，提升了定值结果的可靠性。同时，通过考察药物在毛发中的渗透动力学并确定最佳浸泡时间，解决了现有标准物质制备中药物-角蛋白结合态模拟不足的难题。本工作建立的方法及制备的标准物质为法医毒理学中多类毒品滥用监测和实验室质量控制提供了可靠的工具。

## Abstract (en)

Drug abuse is a major global public health problem. Fentanyl analogs and amphetamine-type stimulants （ATS） are widely abused worldwide. Hair analysis can trace drug use history over months. This technique has unique advantages in forensic toxicology. However， its application is limited by a lack of matrix-certified reference materials （CRMs）. At present， the existing CRMs are unable to simultaneously cover both fentanyl analogs and amphetamine-type stimulants. This study established a hair sample preparation method based on a water‑dimethyl sulfoxide‑hydrochloric acid soaking approach， targeting 15 analytes （nine fentanyl analogs and six ATS）. The soaking time was systematically investigated. Drug incorporation into hair reached a maximum at 24 days. A slight decrease was observed at 25 days. This decrease may result from desorption and solvent-induced structural changes in keratin. Therefore， 24 days was selected as the optimal soaking time. Preparation experiments demonstrated that 14 of the 15 drugs were successfully embedded into the hair matrix. This study also applied a dual-platform quantitative strategy for certification. The two platforms were liquid chromatography-tandem mass spectrometry （LC-MS/MS） and gas chromatography-mass spectrometry （GC-MS）. Cross-validation between the two platforms improved the reliability of certified values. Sample pretreatment conditions were optimized using Box-Behnken response surface methodology. Four factors were investigated： extraction temperature， extraction time， liquid-to-solid ratio， and hydrochloric acid concentration. The effects of the four factors on extraction efficiency followed a decreasing order： extraction temperature>extraction time>liquid-to-solid ratio>hydrochloric acid concentration. The optimal extraction conditions were as follows： extraction temperature 40 ℃， extraction time 50 min， liquid-solid ratio 100∶1 （mL/g）， and hydrochloric acid concentration 0.01 mol/L. Method validation was performed according to international guidelines. For LC-MS/MS， the limits of detection （LODs） and quantification （LOQs） were 0.05 pg/mg and 0.25 pg/mg， respectively. For GC-MS， the LODs and LOQs were 0.02 ng/mg and 0.08 ng/mg， respectively. Good linearities were obtained for all 15 drugs. Correlation coefficients （*r*） were greater than 0.999 for both platforms. Good linearities were obtained for all 15 analytes on both platforms with correlation coefficients >0.999 over the respective concentration ranges Intra-day precisions （*n*=6） were evaluated as relative standard deviations （RSDs）. Inter-day precision （*n*=30 over 5 days） was also calculated. For 14 drugs （except amphetamine）， RSD values ranged from 0.2% to 9.4%. All RSDs were below 10%， indicating good repeatability and intermediate precision. Stability of hair extracts was assessed at 1， 24， 48， 72， and 96 h at room temperature. The extracts were stable for at least 96 h when stored at room temperature， with RSDs below 6.0% for LC-MS/MS and below 10% for GC-MS. Recovery tests were performed at three quality control levels （low， medium， high）. For LC-MS/MS， average recoveries ranged from 84.46% to 116.4% （*n*=3）. For GC-MS， average recoveries ranged from 85.50% to 116.4% （*n*=3）. All RSDs were below 15%. Matrix effects （MEs） were evaluated by comparing matrix-matched standards with solvent standards. ME values between 80% and 120% （*n*=3） were observed for both platforms. These results indicate negligible ion suppression or enhancement. Homogeneity of the prepared hair reference material was assessed. The results show that the uniformity of the hair samples is good. The contents of the 14 drugs in the prepared hair samples were determined. For LC-MS/MS， the values ranged from 1.012 ng/mg to 7.830 ng/mg. For GC-MS， the values ranged from 1.087 ng/mg to 7.712 ng/mg. Amphetamine was not detected by either platform， likely due to its high polarity and weak binding to keratin. Most drugs showed good agreement between the two platforms. Relative deviations were below 10% for most analytes. However， methamphetamine showed a deviation of -12%. This may be due to matrix effects in LC-MS/MS. The GC-MS results for methamphetamine were more robust. The dual-platform approach allowed cross-validation and enhanced the reliability of the certified values. In conclusion， a dual-platform method based on LC-MS/MS and GC-MS was successfully applied to certify a new hair matrix reference material. The cross-validation strategy enhances the accuracy and traceability of the certified values. This work provides a valuable tool for quality control in forensic toxicology laboratories. The prepared reference material and validated method will facilitate inter-laboratory comparisons. They will also support the monitoring of drug abuse in forensic and clinical settings.

药物滥用严重威胁全球公共卫生安全。联合国毒品和犯罪问题办公室（UNODC）发布的《2025年世界毒品报告》显示，2023年全球吸毒人数达3.16亿，其中合成阿片类药物芬太尼及其类似物与苯丙胺类兴奋剂（amphetamine-type stimulants，ATS）的滥用呈现指数级增长，两类药物均造成严重的致死性健康危害^［1］^。芬太尼类物质因具有高效价、合成路径隐蔽、结构易修饰等特点，已成为第3代新型精神活性物质的主要代表^［2］^。ATS如甲基苯丙胺（methamphetamine，MA）具有强烈的中枢神经兴奋作用，在青少年群体中滥用现象严重；ATS的滥用会造成急性精神障碍与心血管疾病等健康风险^［3］^，对该类毒品的精准检测是禁毒工作的关键。

血液、尿液与毛发是目前药物滥用监测中最常用的3种生物检材，在检测窗口、采样便捷性及用药史追溯能力等方面各有特点。血液分析和尿液分析可以提供短期滥用信息，但受限于代谢窗口期（通常≤72 h），难以追溯长期用药史^［4］^。相较之下，毛发作为一种非常规的生物检材具有独特的优势。由于药物及其代谢物可稳定结合于角蛋白基质，毛发分析可提供长达数月的用药记录^［5］^。与血液和尿液分析相比，毛发分析具有简单、无创、药物检测窗口长以及基质稳定的特点。毛发分析常用于法医毒理学、临床药物监测及滥用药物筛查等领域^［6］^，但其广泛应用仍受限于痕量药物检测难度大、基质复杂等若干技术挑战。

为应对毛发分析中的挑战，检测技术的选择与优化至关重要。一方面，前处理技术的革新对于提升方法性能至关重要。符合绿色化学原则的微型化萃取技术（如中空纤维液相微萃取）能有效富集痕量目标物，已成功应用于毛发中苯丙胺类兴奋剂的检测^［7］^。加压液体萃取和在线固相萃取则通过自动化提高了处理效率与通量^［8］^。质谱成像等原位技术，更可在无需提取的情况下直观呈现药物在单根毛发中的空间分布，为推断用药史提供了全新视角^［9］^。另一方面，检测技术各具优势与局限，需根据分析物特性与检测目标合理选择。毛发分析领域常用的检测手段主要包括色谱法、毛细管电泳法、气相色谱-质谱法（GC-MS）与液相色谱-串联质谱法（LC-MS/MS）等。传统色谱法虽分离性能良好，但检测灵敏度有限，难以应对毛发中痕量药物的准确定量需求。毛细管电泳法具备高分离效率与低样品消耗的优点，但其灵敏度与重现性在复杂基质分析中仍面临挑战。GC-MS与LC-MS/MS是目前毛发分析中应用较广泛的联用技术。GC-MS凭借其高选择性和优异的分离能力，适用于挥发性和热稳定性好的化合物（如苯丙胺类兴奋剂）的准确定量与确证。例如，针对依托喹酮等痕量精神药物，已建立灵敏度达pg/mg级别的GC-MS方法^［10］^。然而，GC-MS对强极性、难挥发或热不稳定的化合物（如多数芬太尼类似物）的直接分析能力不足，通常需要烦琐的衍生化步骤，这限制了其覆盖范围和分析通量。相比之下，LC-MS/MS因其无需衍生化即可分析极性化合物、灵敏度高（可达fg/mg~pg/mg级），检测窗口宽，已成为毛发中滥用药物分析的“金标准”，其成熟的验证体系（精密度、准确度、回收率、基质效应等）保障了实验室间的结果可比性与溯源性^［11］^。但LC-MS/MS在复杂毛发基质中易受基质效应干扰，可能导致定量偏差，且常规前处理流程难以获取药物在毛发中的空间分布信息，步骤烦琐、耗时较长。

近年来，高分辨质谱（HRMS），如液相色谱-四极杆飞行时间质谱和液相色谱-静电场轨道阱质谱技术在非靶向筛查、代谢物鉴定及多类物质同步分析中展现出显著优势，可实现对数百种新精神活性物质的同时检测^［12，13］^。然而，HRMS在痕量水平的绝对灵敏度以及宽动态范围的定量精密度方面通常不及三重四极杆质谱。同时，其海量数据的解析高度依赖谱库质量和专业人员经验，且同样无法从根本上解决区分药物摄入与外部污染、独立区分同分异构体等核心难题。

毛发分析在实际应用中面临的一个关键瓶颈是缺乏能够真实模拟药物-角蛋白结合状态的生物基质标准物质^［14］^。尽管国际上已有少数毛发基质标准物质可供使用，但目标物覆盖范围与类型存在局限。例如，美国国家标准与技术研究院（NIST）研制的毛发标准物质SRM 2379（苯丙胺类兴奋剂）和SRM 2380（阿片类药物）分别针对单一类别药物进行定值^［15-17］^。然而，在法医毒理学领域中，多药物滥用（特别是芬太尼类似物与苯丙胺类兴奋剂混用）现象极为普遍。现有标准物质无法在同一基质中同步提供这两类结构和化学性质差异巨大的药物的准确量值，影响了不同实验室间毛发分析结果的可比性与计量溯源性。

基于此，本研究选择了国内外毒品市场中滥用严重、化学性质差异较大的15种药物作为研究对象，旨在研制一种能同时涵盖阿片类（以芬太尼类似物为代表）与兴奋剂（以苯丙胺类兴奋剂为代表）这两大类主要滥用药物的基质标准物质。本工作采用水-二甲基亚砜-盐酸混合溶液浸泡法，制备了模拟毛发中药物与角蛋白真实结合状态的毛发基质标准物质^［18］^，建立了一种适用于多类毒品同步分析的双平台协同检测与验证策略，通过多仪器独立测定与数据互证，确保检测结果的准确性与溯源性，为毛发基质中多类毒品的准确定量与标准物质研制提供了有效解决方案。

## 1 实验部分

### 1.1 仪器、试剂与材料

Waters TQS液相色谱-三重四极杆质谱联用系统（美国Waters公司），配备电喷雾电离源（ESI）和Acquity UPLC超高效液相色谱仪。Agilent 7890A气相色谱仪与7000系列三重四极杆质谱仪联用系统（美国安捷伦公司），配有电子轰击电离源（EI）。Mettler Toledo XP205分析天平（瑞士，最大允许误差为±0.01 mg）。Hitachi CF16RXII高速冷冻离心机（日本）。MM 400球磨机（德国Retsch公司）。

四氢呋喃芬太尼（99.9%）、3，4-亚甲二氧基乙基苯丙胺（3，4-methylenedioxyethylamphetamine，MDEA，99.8%）、替苯丙胺（tenamfetamine，MDA，99.9%）、苯丙胺（amphetamine，AP，99.0%）、去甲芬太尼（99.7%）、甲基苯丙胺-d5（99.8%）购自上海市刑事科学技术研究院。乙酰芬太尼（GBW（E）091075，99.6%）、异丁酰芬太尼（GBW（E）091077，99.8%）、对氟芬太尼（GBW（E）091074，99.7%）、奥芬太尼（GBW（E）091078，99.7%）、硫代芬太尼（GBW（E）091073，99.6%）、4-氟丁酰芬太尼（GBW（E）091076，99.7%）、芬太尼（GBW（E）091009，99.8%）、MA（GBW（E）090151，99.8%）、麻黄碱（ephedrine，GBW（E）090059，99.8%）、3，4-亚甲二氧基甲基苯丙胺（3，4-methylenedioxymethamphetamine， MDMA，GBW09246，99.8%）购自中国计量科学研究院。甲醇、二甲基亚砜、丙酮、乙腈、乙酸铵（以上均为色谱纯）、盐酸（37%浓度）和十二烷基硫酸钠（>99.0%）购自Merck公司。甲酸（色谱纯）购自Thermo Fisher Scientific公司。实验用水为经Milli-Q纯水系统制备的超纯水（电阻率18.2 MΩ·cm）。

本研究使用的毛发样本均为健康成年人理发后丢弃的毛发，采集自理发店，样本提供者均为匿名，不涉及任何个人信息，也未对提供者进行任何形式的干预或随访。研究仅对废弃毛发进行药物分析，不涉及人体生物医学研究中的诊断、治疗或生理功能评估。所有毛发样本均来自女性志愿者，头发为黑色，且无染发或烫发史。

### 1.2 溶液配制

准确称量约10 mg样品，以甲醇为溶剂配制高含量标准储备液（约100 μg/mg）。标准储备液避光保存在0~8 ℃的冰箱中，有效期为3个月。实验过程中，以甲醇为溶剂，按分析要求将标准储备液逐级稀释至5.0~0.01 ng/mg。

### 1.3 毛发样本的制备和前处理过程

首先配制含有15种目标物的混合溶液。分别精密称取约30 mg的乙酰芬太尼、异丁酰芬太尼、对氟芬太尼、奥芬太尼、硫代芬太尼、4-氟丁酰芬太尼、四氢呋喃芬太尼、芬太尼、MA、MDMA、MDEA、MDA及麻黄碱，以及约5 mg的去甲芬太尼与AP，置于1 000 mL容量瓶中。向容量瓶中加入约800 mL水-二甲基亚砜（DMSO）（体积比为1∶1）混合溶液（含0.02 mol/L盐酸），振荡使标准品充分溶解，用该混合溶液定容至刻度，混合均匀。取约40 g经清洗后（用质量浓度为1.0 g/L的十二烷基硫酸钠溶液、水、丙酮依次洗涤并干燥）的空白毛发样本，完全浸没于上述混合溶液中，于室温、避光条件下静置浸泡24天，以确保药物通过扩散作用充分结合至毛发角蛋白基质中。浸泡结束后弃去浸泡液。为去除表面残留的溶剂及未结合的游离药物，对毛发样本进行洗涤。将毛发完全浸没于色谱级甲醇中（液固比为20∶1， mL/g），置于超声清洗器中，在功率100 W、频率40 kHz下超声洗涤5 min，弃去洗涤液，重复4次。保留第四次洗涤液，用于后续评估洗涤效率，确保无目标物检出（信号低于方法检出限），以证明药物已稳定结合于毛发基质内部。第四次洗涤液中目标物信号均低于本方法在毛发基质中的检出限，表明残留药物浓度较低，不影响后续测定结果。清洁后的毛发样本在室温条件下晾干24 h，随后转移至真空干燥箱中持续脱水48 h。经充分干燥的样本使用球磨机进行粉碎处理（粒径<0.5 mm），并通过混合仪确保均匀性，最后分装，于室温、避光环境中稳定保存^［19］^。毛发样本的前处理过程如图1所示，在毛发样本中加入甲基苯丙胺-d_5_内标（internal standard，IS）溶液，超声提取50 min，取上清液1.5 mL，于40 °C下氮气吹干，残留物以200 µL初始流动相复溶，经14 000 r/min（约18 200 g）离心5 min，取上清液过0.22 µm聚四氟乙烯滤膜，分别注入LC-MS/MS和GC-MS系统进样分析。

**图1:**
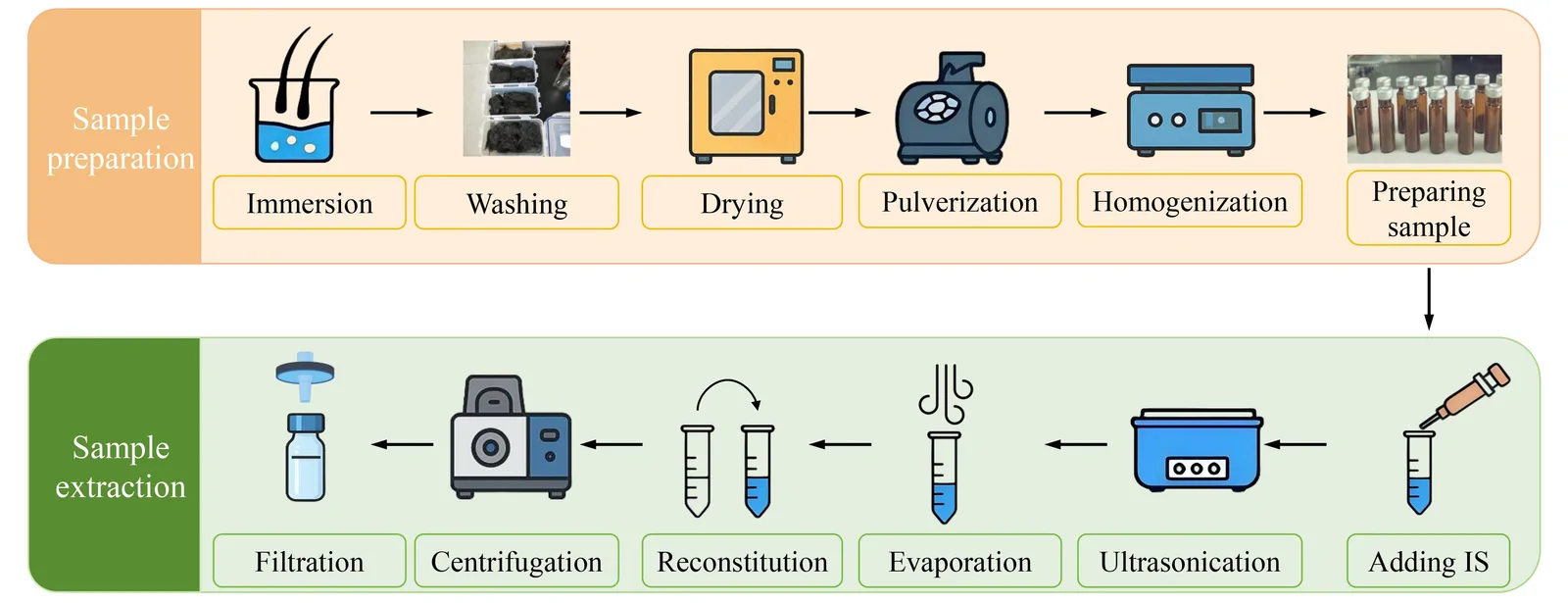
毛发样本的制备和前处理流程图

### 1.4 LC-MS/MS和GC-MS分析条件

LC-MS/MS方法 参考文献［^19^］建立的9种芬太尼类似物的LC-MS/MS分析方法，并在此基础上将目标物扩展至9种芬太尼类似物和6种苯丙胺类兴奋剂。色谱柱：Acquity UPLC HSS T_3_（100 mm×2.1 mm，1.8 μm，Waters公司）；流动相A：0.01 mol/L乙酸铵水溶液（含0.1%甲酸）；流动相B：乙腈；色谱分离采用梯度洗脱程序：0~4 min，15%B~30%B；4~10 min，30%B~45%B；10~15 min，45%B~95%B；15~17 min，95%B；17~17.1 min，95%B~15%B；17.1~20 min，15%B。流速为0.2 mL/min，色谱柱温度30 ℃，进样量2 μL。质谱检测在电喷雾正离子模式（ESI^+^）下进行，离子源温度为200 ℃，毛细管电压为1.58 kV。脱溶剂气温度与流速分别为650 ℃和800 L/h，锥孔气流速为150 L/h，检测采用多反应监测（multiple reaction monitoring， MRM）模式。其他参数见表1。

**表1:** LC-MS/MS中芬太尼类似物和苯丙胺类兴奋剂的质谱参数

Drug name	Retention time/ min	*M*_r_	Precursor ion （*m/z*）	Product ion （*m/z*）	Cone voltage/V	Collision energy/V
Norfentanyl	5.36	232.32	233.25	84.25^*^	82	18
Acetylfentanyl	8.75	322.45	323.31	188.29^*^	10	20
				105.23	10	32
Ocfentanil	11.74	370.47	371.33	188.29^*^	10	24
				105.23	10	34
Thiofentanyl	13.11	342.50	343.23	194.27^*^	16	20
				111.21	16	46
Fentanyl	9.59	336.48	337.34	188.29^*^	4	20
				105.23	4	34
4-Fluorofentanyl	10.35	354.47	355.33	188.29^*^	8	26
				105.23	8	34
Isobutyrylfentanyl	8.81	350.51	351.37	188.29^*^	16	24
				105.23	16	34
4-Fluorobutyrylfentanyl	14.38	368.50	369.36	188.29^*^	6	24
				105.23	6	36
Tetrahydrofuranylfentanyl	15.25	378.52	379.29	188.29^*^	94	38
				105.23	94	22
Amphetamine （AP）	1.35	135.00	137.10	92.15^*^	55	25
				119.00	55	15
Methamphetamine （MA）	2.74	149.23	148.24	133.24^*^	64	18
				117.26	64	16
3，4-Methylenedioxyethylamphetamine （MDEA）	5.17	207.00	208.22	105.23^*^	28	22
				132.85	28	24
3，4-Methylenedioxymethamphetamine （MDMA）	4.44	194.00	194.22	163.27^*^	32	12
				105.23	32	22
Tenamfetamine （MDA）	4.00	179.20	181.10	135.00	70	26
				164.20^*^	70	13
Ephedrine	2.72	148.00	148.16	114.91	42	16
				133.24^*^	42	16

* Quantitation ion.

GC-MS方法 参考本课题组^［20］^前期建立的苯丙胺类兴奋剂GC-MS分析方法。色谱柱：DB-5MS（30 m×0.25 mm×0.25 μm）；程序升温条件：初始温度80 ℃，保持1 min，以10 ℃/min的速率升温至250 ℃，保持2 min，再以10 ℃/min的速率升温至300 ℃，保持5 min。进样参数：进样口温度280 ℃，进样体积1 μL，分流比10∶1；载气与流速：氦气，1.0 mL/min；溶剂延迟：5.0 min。质谱分析在EI源下进行，电子能量为70 eV，离子源与传输线温度分别设定为230 ℃与250 ℃。定量分析采用选择离子监测（selected ion monitoring，SIM）模式，GC-MS中芬太尼类似物和苯丙胺类兴奋剂的特征离子见表2。

**表2:** GC-MS中芬太尼类似物和苯丙胺类兴奋剂的特征离子

Drug name	Characteristic ions （*m*/*z*）	Retention time/min
Norfentanyl	83^*^， 120， 159， 175	15.84
Acetylfentanyl	231^*^， 146， 188	20.59
Ocfentanil	279^*^， 176， 280	24.19
Thiofentanyl	245^*^， 146， 93	23.22
Fentanyl	245^*^， 146， 189， 280	22.89
4-Fluorofentanyl	263^*^， 164， 220	21.21
Isobutyrylfentanyl	259^*^， 146， 189	21.92
4-Fluorobutyrylfentanyl	164^*^， 277， 207	23.74
Tetrahydrofuranylfentanyl	287^*^， 189， 146， 158	24.86
AP	44^*^， 91， 117， 65	6.18
MA	58^*^， 91， 56	6.18
MDEA	72^*^， 135， 136， 77	10.78
MDMA	58^*^， 77， 59， 135	10.08
MDA	44^*^， 136， 77， 51	10.18
Ephedrine	58^*^， 77， 105	8.65

* Quantitation ion.

## 2 结果与讨论

### 2.1 毛发样本制备中浸泡时间的选择

为探究药物在毛发中的渗透动力学，实验于第7、15、20、22、24和25天共6个时间点采集经DMSO混合溶液浸泡的毛发样本，每个时间点取3份毛发样本采用LC-MS/MS进行测定。图2展示了药物含量随时间变化的趋势，反映出药物在毛发中的积累过程。结果显示，药物累积过程可分为3个阶段：（1）第7~22天：初始渗透期，药物含量逐步上升；（2）第24天：饱和期，此时进入头发中的药物浓度最高；（3）第25天：下降期，药物含量下降。

**图2:**
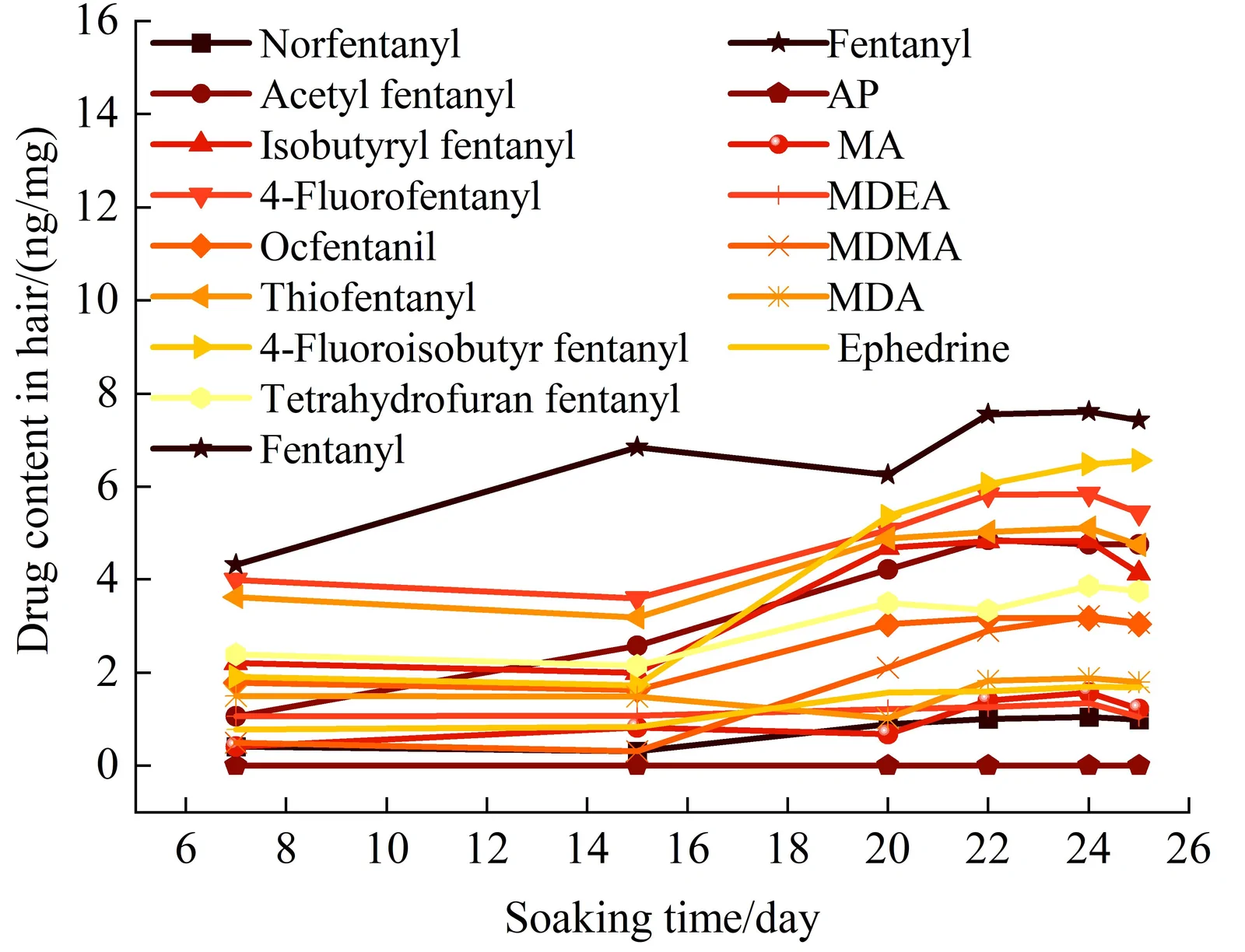
不同浸泡时间内毛发中15种目标物的含量

在本实验条件下，浸泡24天时毛发中药物含量达到观察期内的最高值，提示该时间点接近药物-角蛋白结合的动态平衡。该结果为毛发基质标准物质的制备工艺标准化提供了关键依据。本实验中观察到的药物在毛发中的渗透动力学与药物-毛发相互作用的基本机制相符。毛发作为一种复杂的生物基质，其主要成分角蛋白富含多种极性基团和结合位点，对外源性药物的吸附能力存在理论上的上限。在本实验中，第25天毛发中的药物含量下降，可能是物理解吸附与溶剂效应共同作用的结果。长期暴露于强极性溶剂DMSO中可能引起毛发中角蛋白网格结构改变，促使部分已结合的药物释放到溶液中。研究表明DMSO能显著影响分子扩散行为与基质稳定性，Kintz等^［21］^发现DMSO比*N*-甲基吡咯烷酮具有更快的溶剂交换与药物扩散速率。DMSO的这种特性在浸泡初期可以有力地促进药物渗透，但在后期可能会加速药物解吸附。另一方面，角蛋白中有限结合位点的饱和也是关键因素。当药物吸附达到饱和状态后，药物的掺入与释放之间会形成动态平衡，该现象符合吸附等温线特征。角蛋白中的极性基团（如羟基、氨基等）可通过氢键等分子间作用力固定药物分子，当可用位点耗尽时，新进入的药物分子难以稳定结合，最终在溶液-基质界面形成动态平衡，从而在表观上呈现含量下降。

### 2.2 样品前处理条件的优化

采用Box-Behnken响应面法，系统考察了提取温度（*A*）、提取时间（*B*）、液固比（*C*）、盐酸浓度（*D*）等条件对药物回收率的影响（图3）。采用Design Expert 13.0软件设计四因素三水平试验，探索毛发中提取药物的最佳条件。以甲基苯丙胺为例，拟合药物含量（ng/mg）与各提取条件的二次多元回归方程如下：

**图3:**
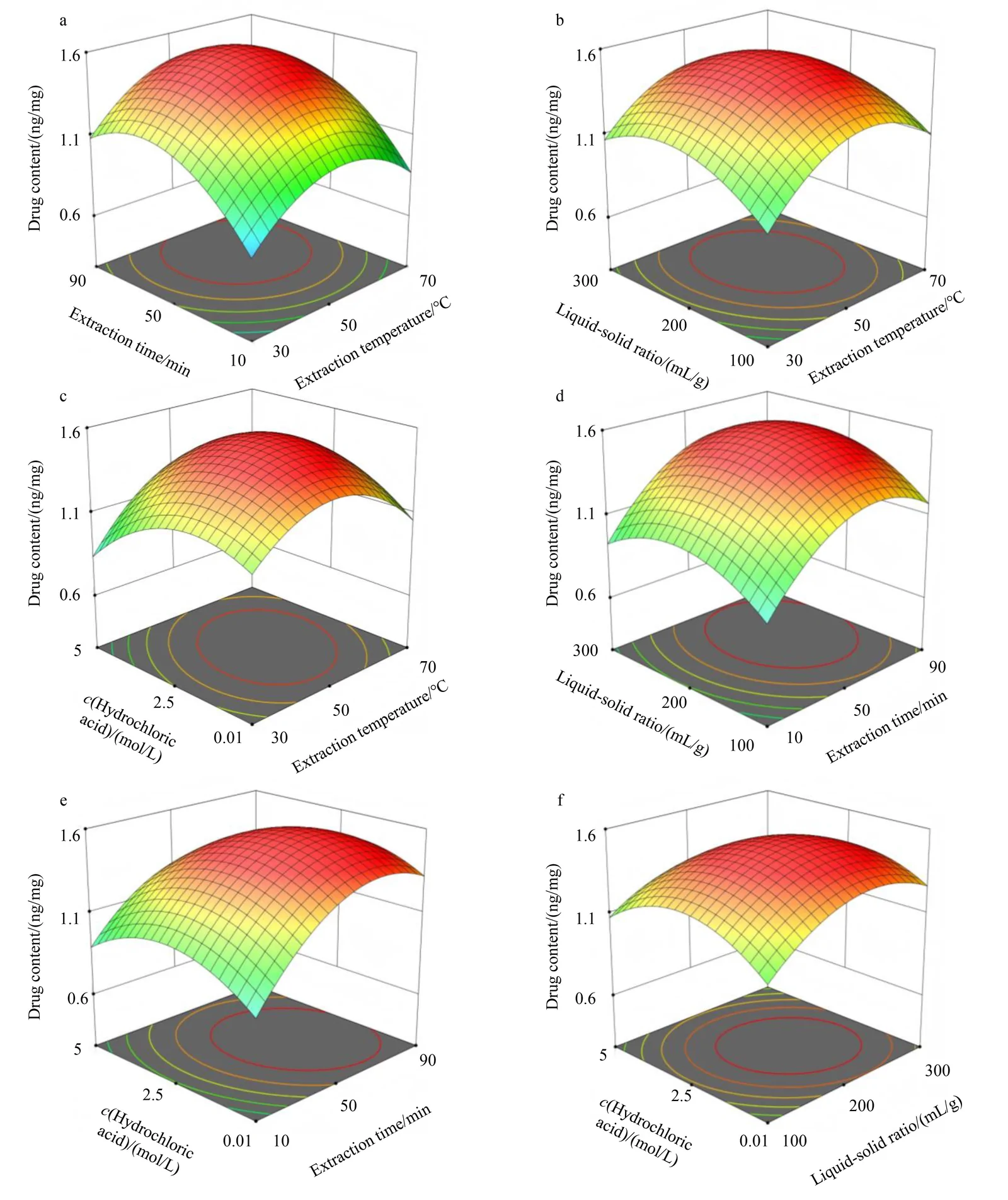
不同提取参数对甲基苯丙胺含量的影响

药物含量（ng/mg）=1.56-0.215 3*A*+0.062 9*B*-0.090 3*C*+0.084 4*D*-0.076 2*AB*+0.020 9*AC*+0.066 9*AD*-0.027 3*BC*+0.064 1*BD*+0.200 7*CD*-0.259 2*A*^2^-0.324 5*B*^2^+0.046 9*C*^2^+0.071 0*D*^2^。

模型的*F*值为3.97（*p*<0.05），表明该模型显著。在这种情况下，*A*、*CD、A*^2^和*B*^2^是重要的模型项。各因素对毛发中15种药物提取量的影响为提取温度>提取时间>液固比>盐酸浓度。响应面与等高线图直观反映了因素间的交互作用，其中椭圆形等高线表明*CD*等因素间存在显著协同效应，而圆形或不规则轮廓则提示其余因素间交互作用较弱^［22］^。

利用Design Expert 13.0软件预测提取温度为38 ℃、提取时间为54 min，液固比为100∶1 （mL/g）、盐酸浓度为0.01 mol/L时，药物含量达到峰值。在此条件下，MA预测值为1.973 ng /mg。实际测定结果显示，在提取温度40 ℃、提取时间50 min、液固比100∶1 （mL/g）、盐酸浓度0.01 mol/L的条件下，MA的测得值为1.915 ng/mg。这证实了响应面模型的有效性。因此，确定毛发中15种药物的最佳提取条件为提取温度40 ℃，提取时间50 min，液固比100∶1 （mL/g），盐酸浓度0.01 mol /L。

### 2.3 方法学验证

检出限（LOD）和定量限（LOQ） 以空白毛发样本为基质，制备了含有15种目标物的低浓度标准工作溶液。LOD和LOQ分别以3倍和10倍信噪比（*S/N*）确定。结果表明，LC-MS/MS的检出限是0.05 pg/mg，定量限是0.25 pg/mg，GC-MS法的检出限是0.02 ng/mg，定量限是0.08 ng/mg。

线性范围 将毛发样本按照前处理方法进行处理，采用内标工作曲线法进行定量分析。每个含量水平平行测定3次。以药物含量（*x，* ng/mg）为横坐标，目标物与甲基苯丙胺-d_5_的峰面积比（*y*）为纵坐标，绘制标准曲线。结果如表3所示，LC-MS/MS中，13种目标物在0.01~0.2 ng/mg范围内表现出良好的线性关系，相关系数（*r*）*>*0.999。AP和MDA在0.06~0.5 ng/mg含量范围内线性关系良好，*r*>0.999。在GC-MS中，15种目标物均在一定范围内线性关系良好，*r>*0.999。两种分析方法均满足定量分析的要求。

**表3:** 15种目标物的线性关系

Drug	GC-MS			LC-MS/MS		
	Linear range/（ng/mg）	Regression equation	*r*	Linear range/（ng/mg）	Regression equation	*r*
Norfentanyl	0.4-4.5	*y*=0.1311*x*+0.1568	0.9998	0.01-0.2	*y*=21.173*x*+0.1193	0.9997
Acetylfentanyl	0.9-4.5	*y*=0.2019*x*+0.3706	0.9996	0.01-0.2	*y*=61.131*x*+0.3503	0.9996
Isobutyrylfentanyl	0.4-4.5	*y*=0.27*x*+0.6094	0.9997	0.01-0.2	*y*=51.327*x*+1.2441	0.9996
4-Fluorofentanyl	0.4-4.5	*y*=0.2233*x*+0.4528	0.9996	0.01-0.2	*y*=71.813*x*+0.0265	0.9995
Ocfentanil	0.4-4.5	*y*=0.2182*x*+0.4271	0.9997	0.01-0.2	*y*=84.916*x*+0.462	0.9995
Thiofentanyl	0.4-4.5	*y*=0.175*x*+0.4698	0.9996	0.01-0.2	*y*=45.938*x*+0.1992	0.9995
4-Fluorobutyrylfentanyl	0.4-4.5	*y*=0.1424*x*+0.3142	0.9997	0.01-0.2	*y*=16.077*x*+3.8265	0.9998
Tetrahydrofuranylfentanyl	0.9-4.5	*y*=0.1195*x*+0.2369	0.9995	0.01-0.2	*y*=31.942*x*+0.0672	0.9997
Fentanyl	0.4-4.5	*y*=0.1425*x*+0.3061	0.9996	0.01-0.2	*y*=56.917*x*+0.1109	0.9999
AP	0.9-4.3	*y*=0.5144*x*+0.9685	0.9996	0.06-0.5	*y*=0.1779*x*+0.0163	0.9995
MA	0.9-4.5	*y*=1.0571*x*+1.0671	0.9998	0.01-0.2	*y*=7.689*x*-0.0435	0.9997
MDEA	0.9-4.4	*y*=1.056*x*+4.1112	0.9996	0.01-0.2	*y*=23.794*x*+0.1312	0.9996
MDMA	0.9-4.4	*y*=0.6487*x*+2.0984	0.9995	0.01-0.2	*y*=38.361*x*+0.12	0.9998
Ephedrine	0.9-4.4	*y*=0.604*x*+0.8299	0.9997	0.01-0.2	*y*=10.336*x*-0.0034	0.9999
MDA	0.9-4.4	*y*=0.3614*x*+0.5927	0.9997	0.06-0.5	*y*=0.0566*x*+0.0062	0.9996

*y*： the ratio of the peak area of the substance to that of methamphetamine-d_5_； *x*： drug content， ng/mg. *r* ： correlation coefficient.

日内和日间精密度 日内精密度通过分析6份平行配制的毛发样本提取液进行评价。每份样品按1.3节的实验条件进行前处理，然后分别注入LC-MS/MS和GC-MS系统进行测定。以此计算6个独立测定结果（*n*=6）的相对标准偏差（RSD），作为方法的日内精密度（重复性）。日间精密度通过在连续5个工作日内，每日独立制备并测定一组6份平行样品（*n*=30）进行评价，计算其RSD。精密度结果汇总于附表1（www.chrom-China.com）。结果表明，除AP外，14种目标物在两种检测方法下，日内与日间精密度的RSD值小于10%，表明所建立的分析方法具有良好的重复性与日间精密度。

稳定性 取制备好的毛发提取液，在室温（25±5） ℃条件下分别放置1、24、48、72和96 h后进样分析，每个时间点平行测定3次。以待测物与甲基苯丙胺-d_5_的峰面积比值计算RSD，评估提取液的稳定性。结果表明，LC-MS/MS中RSD<6%，GC-MS中RSD<10%。制备的毛发提取液在室温下96 h内具有良好的稳定性，稳定性试验结果见附表2。

回收率 为评估整个分析方法的准确度，采用低、中、高（低浓度（LQC）1.0 ng/mg、中浓度（MQC）4.0 ng/mg、高浓度（HQC）8.0 ng/mg（以毛发基质中药物含量计））3个水平的质控样品，进行加标回收试验，每个水平平行测定3次。准确称取9份空白毛发样本（每份约10 mg），分别加入3个水平的15种目标物混合标准溶液及等量的甲基苯丙胺-d_5_。根据回归方程，计算不同加标水平下的回收率。结果表明，LC-MS/MS中，15种药物在LQC、MQC、HQC水平下的平均回收率为96.04%～110.8%、84.46%～104.3%、88.69%～100.7%，RSD<15%。GC-MS中LQC、MQC、HQC的平均回收率分别为96.84%～116.4%、85.50%～102.1%、86.14%～112.9%，RSD<15%。实验结果表明，建立的LC-MS/MS和GC-MS方法具有高准确度。

基质效应（matrix effect，ME） 通过分析低、中、高3个水平的基质匹配标准溶液（由空白毛发提取液配制）与纯甲醇标准溶液的峰面积之比来评价基质效应。每个浓度水平平行配制3份基质匹配标准溶液和3份纯甲醇标准溶液，每份溶液进样测定3次，分别计算每个浓度水平下的RSD。ME=（*A*/*B*）×100%，*A*为样品在毛发基质中的峰面积；*B*为样品在甲醇中的峰面积；ME>100%表示存在离子增强，ME<100%表示存在离子抑制。结果表明，LC-MS/MS和GC-MS中15种目标物的基质效应在80%～120%范围内，RSD<15%，符合分析要求。15种目标物的回收率与基质效应结果见附表3。

### 2.4 样本分析

从分装的200瓶毛发样品中随机抽取11瓶，每个样品测定3次，其LC-MS/MS MRM色谱图和GC-MS SIM色谱图分别见图4和图5。LC-MS/MS和GC-MS的定量结果见表4，AP在两种方法中均未检出，第四次洗涤液的检测结果显示AP信号低于方法检出限，推测其强极性导致与角蛋白结合较弱，在洗涤过程中被优先洗脱。LC-MS/MS测得制备的毛发样本中14种目标物的含量为1.012~7.830 ng/mg，GC-MS测得制备的毛发样本中14种目标物的含量为1.087~7.712 ng/mg，其中多数目标物的相对偏差低于10%，表明两种方法在定量方面具有较高的可比性与重现性。然而，MA（偏差-12%）在两种方法间存在一定差异，可能与其在LC-MS/MS中受基质效应影响较大有关（见附表3），而GC-MS基质效应相对较小，定量结果更为稳健。

**图4:**
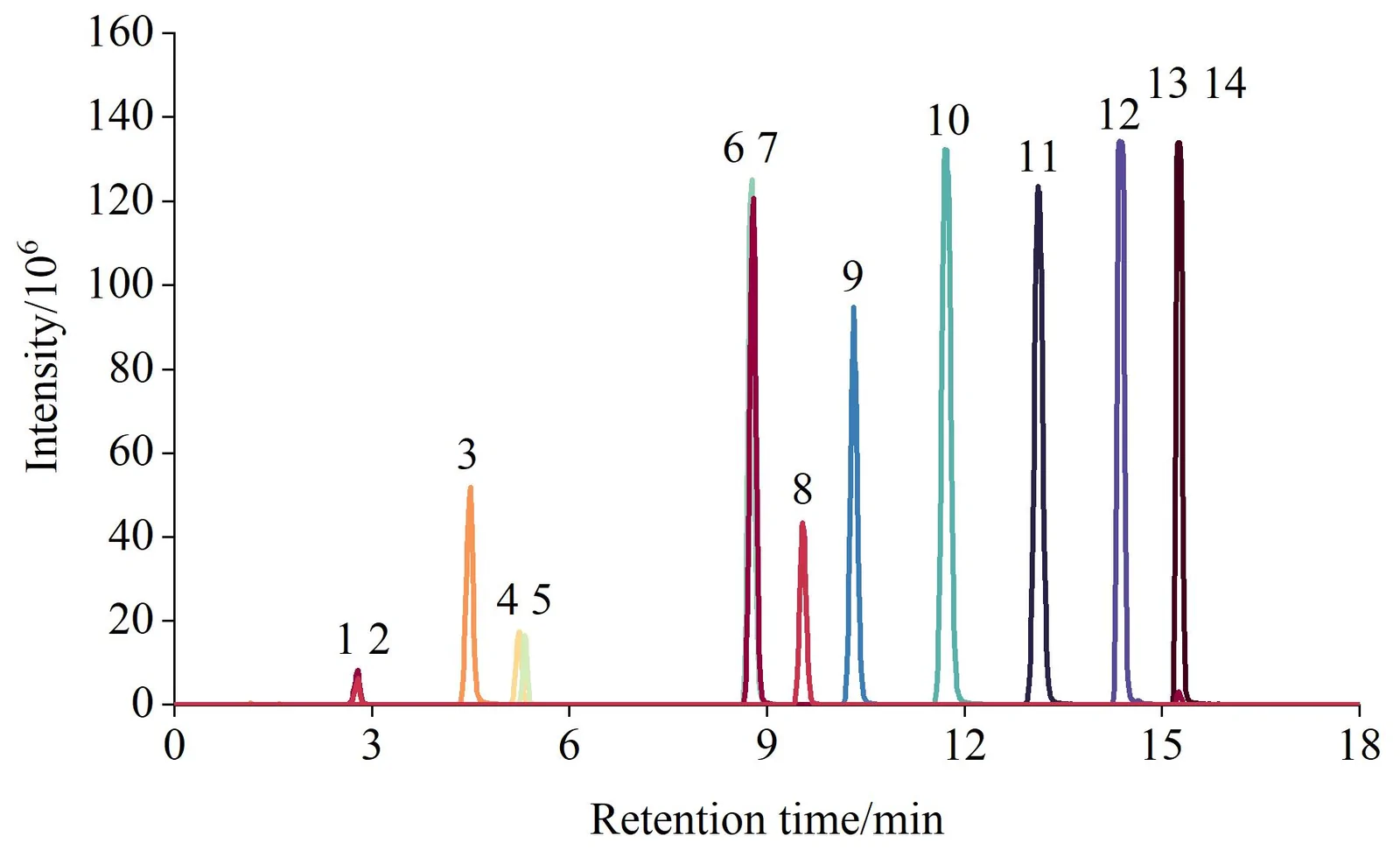
毛发中15种目标物的LC-MS/MS色谱图

**图5:**
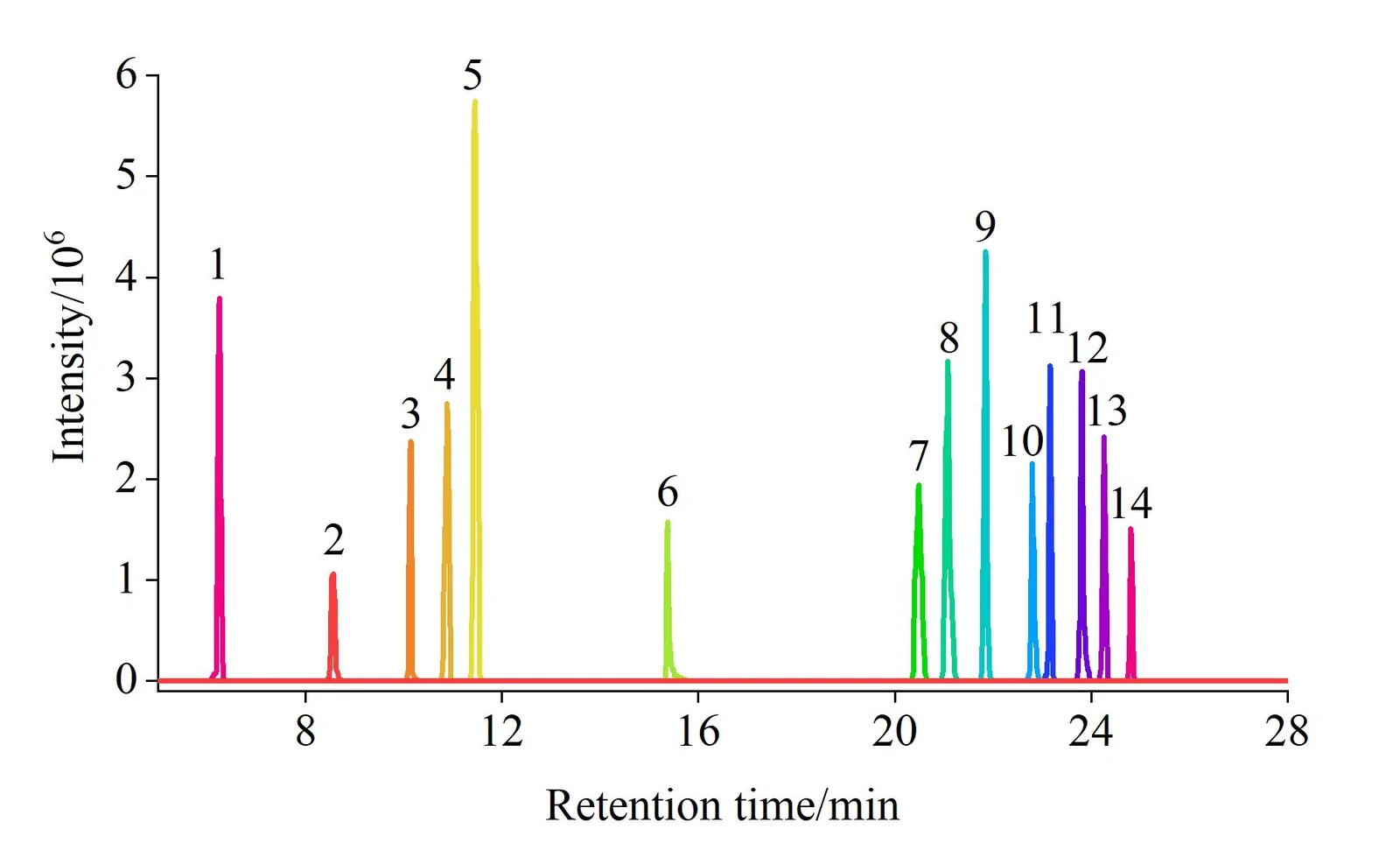
毛发中15种目标物的GC-MS色谱图

**表4:** LC-MS/MS与GC-MS结果比较

Drug name	LC-MS/MS （*n*=3）		GC-MS （*n*=3）		Deviation/%	Average value/（ng/mg）
	Average value/（ng/mg）	RSD/%	Average value/（ng/mg）	RSD/%		
Norfentanyl	1.012	1.6	1.087	3.7	-7.1	1.050
Acetylfentanyl	4.788	0.6	4.821	2.0	-0.7	4.805
Isobutyrylfentanyl	4.826	0.3	4.816	1.9	0.2	4.821
4-Fluorofentanyl	5.830	0.2	5.786	1.6	0.8	5.808
Ocfentanil	3.188	1.1	3.031	2.2	5.1	3.110
Thiofentanyl	5.010	0.6	4.925	2.0	1.7	4.968
4-Fluorobutyrylfentanyl	6.303	0.4	6.028	1.7	4.5	6.166
Tetrahydrofuranylfentanyl	3.444	0.2	3.451	3.0	-0.2	3.448
Fentanyl	7.830	0.3	7.712	1.9	1.5	7.771
AP	-	-	-	-	-	-
MA	1.789	1.3	2.015	2.0	-12	1.902
MDEA	1.321	0.3	1.322	1.2	-0.1	1.322
MDMA	3.284	0.7	3.325	1.2	-1.2	3.305
Ephedrine	1.667	1.3	1.667	1.6	0	1.667
MDA	1.938	1.6	2.112	2.7	-8.6	2.025

-： not detected. Deviation=［（LC-MS/MS result-GC-MS result）/average value ］×100%； average value=（LC-MS/MS result+GC-MS result）/2.

从标准物质的200个包装中随机抽取11个包装，每个包装取3个子样进行瓶间和瓶内均匀性检验。最小取样量为1 mg。按照1.4节所述的LC-MS/MS方法进行测定。将均匀性方差分析结果，即瓶间标准偏差（*s*_bb_）与该特性量值不确定度的预期目标进行比较，若不显著，则标准物质视为均匀。采用方差分析法（analysis of variance， ANOVA）进行均匀性研究结果的评价，毛发中14种目标物均匀性检验结果见附表4。*F*检验结果表明，LC-MS/MS中，14种目标物的*F*_测得值_<*F*_0.05_（10，22）=2.30，说明制备的毛发样本均匀性良好。

## 3 结论

本研究以水-二甲基亚砜-盐酸混合溶液浸泡法制备了含14种目标药物的毛发基质标准物质候选物，通过系统考察药物渗透动力学，确定24天为最佳浸泡时间，实现了14种目标药物在毛发基质中的稳定结合。在此基础上，建立了LC-MS/MS与GC-MS双平台定量分析方法，方法学验证结果表明准确度和精密度良好，满足毛发痕量分析的定量要求。LC-MS/MS对芬太尼类似物灵敏度更优，GC-MS在苯丙胺类兴奋剂分析中基质干扰更小，双平台交叉验证策略有效提升了定量结果的可靠性^［23］^。本研究中药物在毛发中的渗透动力学与Kintz等提出的被动扩散模型一致^［24，25］^。制备的毛发基质标准物质候选物均匀性良好，可为法医毒理学实验室质量控制提供可靠工具。

本研究尚存在以下不足：（1）内源性干扰物可能会影响药物结合效率。所用毛发样本均来自成年女性，颜色为黑色，且无染发或烫发史，未来需进一步探究不同发质对药物结合的影响^［26，27］^；（2）代谢物在滥用药物诊断中具有重要意义，但本研究未完全覆盖芬太尼类似物和苯丙胺类兴奋剂的代谢物^［28］^；（3）AP未检出，后续将聚焦于制备工艺的优化（如改变浸泡溶剂配比、延长浸泡时间等），并将更多新型精神活性物质纳入检测范围，结合高分辨质谱技术实现非靶向筛查。同时，引入磁固相萃取、微流控芯片等先进前处理技术提升检测效率^［29，30］^。联合多中心实验室验证标准物质的适用性，建立跨平台数据比对体系，结合代谢通路分析探索药物滥用标志物的联合检测策略^［31］^。
